# Hyper-ambition and the Replication Crisis: Why Measures to Promote Research Integrity can Falter

**DOI:** 10.1007/s10805-024-09528-5

**Published:** 2024-04-03

**Authors:** Yasemin J. Erden

**Affiliations:** Philosophy, Faculty of Behavioural, Management and Social Sciences (BMS), University of Twente, Enschede, Netherlands

**Keywords:** Replication crisis, Science correction, Research integrity, Values, Motivations, Ambition, hyper-ambition

## Abstract

This paper introduces the concept of ‘hyper-ambition’ in academia as a contributing factor to what has been termed a ‘replication crisis’ across some sciences. The replication crisis is an umbrella term that covers a range of ‘questionable research practices’, from sloppy reporting to fraud. There are already many proposals to address questionable research practices, some of which focus on the values, norms, and motivations of researchers and institutes, and suggest measures to promote research integrity. Yet it is not easy to promote integrity in hyper-competitive academic environments that value high levels of ambition. I argue that in such contexts, it is as likely that a kind of hyper-ambition is fostered that (inadvertently or otherwise) prioritises individual success above all, including to the detriment of scientific quality. In addition, efforts to promote values like integrity falter because they rely on sufficient uniformity in motivations or tendencies. Codes and guidance promoting integrity are, however, likely to influence those for whom such values are not optional, while others simply find ways around them. To demonstrate this I offer a thought experiment in which we consider the imaginary working situations of two ordinary academics. I conclude that tackling questionable research practices in the light of the replication crisis requires robust ‘top down’ measures that expect and accommodate a broader range of academic values, motivations, and tendencies, while challenging those that help to promote hyper-ambition.

## Introduction Part One: The Story of Toni and Sophie

I begin this paper with a thought experiment. A thought experiment is a kind of hypothetical case study that is common in philosophy. These hypothetical accounts provide fictional, or fictionalised scenarios that support the exploration of philosophical and concrete questions relevant to an argument. In this way, thought experiments offer scope for the identification and thereby analysis of the reasoning in an argument, as well as any implicit or explicit assumptions and intuitions (Brun, 2017). While some thought experiments cover extraordinary, fantastic, or science fiction accounts of the world, this is not my aim here. Instead I present an account of academia that (I hope) is familiar and quite ordinary, and with what I consider to be some unexceptional behaviours and actions.

In this example two academics, Toni and Sophie, are broadly at the same career stage: each is somewhat early in their career but with some years of experience, some publications, teaching experience, and with successfully completed postdoctoral contracts behind them. Both are newly tenured. These academics are ostensibly colleagues. They work in the same department, attend the same meetings, see each other at work events, and work in similar areas. But they are also considered competitors. Each has their eye on promotion, which they know will require research proposals, ideally with some success, further publications, conference papers, and invited talks. In this example, what separates these academics amounts to some qualities that can be difficult to measure, for instance, their tendencies, values, and motivations, and, centrally for this paper, how these play out in their behaviour. By *values* I mean what a person or a group consider to be important, including by their actions. By *motivations* I mean that which is used as a reason for action, or which is described as driving an action. By *tendencies* I mean what a person is likely to do or even what they generally do. While these are complex terms in philosophy and psychology, I offer these ordinary definitions so as to tease out some differences between Toni and Sophie.

Toni is highly and primarily ambitious, by which we can (in this case) understand that she seeks reward irrespective of whether the success is merited. Because this is her aim, she prioritises accordingly: she makes time to socialise with people who have power, or those who are working on funding proposals she is interested to join, even if this means she makes less time for other colleagues. Having evaluated what promotion requires, she will devote herself to research as much as she can, even if this is to the detriment of her students. This is not to say that her teaching necessarily suffers: she will do enough so that her teaching evaluations are *fine* (important for promotion), but what she will not do is give much time to students who are struggling. Someone else can deal with them. Sometimes, in the rush to be published, she attends talks (including by Sophie) and uses some of the ideas she hears. She does not credit the people she takes the ideas from, since she thinks that these are public statements and there is nothing stopping her from being ‘inspired’. When work is particularly busy she slacks on some of the less important tasks, especially where senior colleagues are unlikely to notice that her peers have to pick up the slack. After a year, Toni has published three new papers, is being viewed as a promising and ambitious colleague by senior staff, with good promotion prospects.

Sophie wants to do well and achieve her goals, and she is especially keen to receive recognition, by which we can understand that she wants to be rewarded for the quality of her work. Her main aim is to achieve rewards that she has merited. Partly because of this, and partly because she is considerate of others, she spreads her priorities: she makes time for colleagues who are newer than her and who have questions. She gives time to her research, but not to the detriment of her students. She is a good teacher and goes beyond the minimum so as to help her students to thrive. Her teaching evaluations are excellent (important for promotion), but these do not capture the additional work that she does, including making time for students who are struggling. Some students from Toni’s class hear that Sophie is willing to help, so they contact her because they are also struggling. She gives them some of her time as well. The improvement to these students’ lives isn’t captured in any metrics so the work is largely unnoticed, including by colleagues. All of this work impacts on Sophie’s time to prepare her research for publication, but she persists and even gives a department talk on some exciting new ideas she’s developing. None of the senior staff are able to attend, but she is pleased to see many colleagues in the audience, including Toni. She is happy to discuss her ideas, including at the end of the talk when Toni asks her a lot of questions. She is pleased that people are interested. She thinks about how to credit her colleague, John, who asked a particularly insightful question that led her to address a problem she had been puzzling about, and decides to add him to the acknowledgements. Around marking time, when work is busy, she finds that some colleagues are unable to complete important tasks and this means that students will suffer. She feels badly for the students, so agrees to finish the work. After a year, Sophie has struggled to find the time to publish her work, but she does have the paper she presented in draft, and which colleagues at the talk considered quite novel. Senior colleagues are unsure how to understand Sophie’s prospects. Her teaching evaluations are very good, but she seems to have fallen behind in her research. Especially as the apparently novel idea she has been working on has recently been published by her colleague, Toni. Her promise and ambition are questioned by senior staff who are evaluating her promotion prospects.

## Introduction Part Two: Academic Problems

The story of Toni and Sophie is (anecdotally) a familiar one, and it also not an especially surprising one. On the one hand, we see that academic incentives like grants and prizes, titles and promotion, popularity and citation indexes, dominate the measures against which success is evaluated (Ritchie, 2020). We can further see that incentives affect behaviour (Wenaas 2019) especially where these are based in competition (Moore et al., 2017). On the other hand, we also have evidence that prioritising things like novelty over replication can create problems (“Not the first, not the best” 2021), that rankings and citation indexes capture problematic data or use arbitrary standards (Wenaas 2019), and that all of this can encourage overstatements from researchers so as to meet indicators like *excellence* by defining their work as *exceptional* and *outstanding*, which in turn can affect the quality of research (Moore et al., 2017).

There is much evidence that all of this feeds into what has been called a ‘replication crisis’ across a number of sciences, and which has thrown academic measures of success into sharp relief (Ioannidis, 2005; Fanelli, 2009; Ritchie, 2020). Replicability in this context can be understood as a cross-disciplinary umbrella term that spans various types of replication, including computational (Parsons et al., 2022), and which can be affected by context and other variables. Talk of a replication crisis partly arose from a series of large-scale replication projects that resulted in lower than expected success rates (Korbmacher et al., 2023). This includes a major study in psychology in 2015 which undertook replications of 100 studies but found successful results in only 39% of these, thereby highlighting substantial problems in that field (Open Science Collaboration, 2015). As Korbmacher et al. (2023) point out, this rate should have been closer to 89% as a minimum. Other scientific disciplines are following suit in identifying similar issues (Andreoletti, 2020).

The term ‘replication crisis’ is not neutral, and some have sought to reframe the discussion by shifting talk of a ‘crisis’ to a ‘credibility revolution’ (Korbmacher et al., 2023), which is considered to offer a more positive account that is solution-focussed (Parsons et al., 2022). I continue to refer to the replication crisis in this paper, however, partly because it is a relatively well known and thereby commonly used expression, as demonstrated in the literature that I cite, and partly because I think it captures well the urgency that I consider these issues to deserve. That said, I have no particular objection to efforts to reframe the debate.

Many correcting measures from within science have been proposed to address these issues. Some are technical or focus on correcting the processes by which scientific research is undertaken, measured, valued, evaluated, promoted, applied, and understood by the broader community (Ritchie, 2020; Vazire & Holcombe, 2022; Korbmacher et al., 2023). Others focus on the role of gatekeepers like journals, editors, and reviewers, in conjunction with universities, funders, and other stakeholders (Barzilai-Nahon, 2009; Primack et al., 2019). A common theme in these approaches is to explore the values, norms, or motivations that lead to the problems in the first place. Here I argue that a joint ‘bottom up’ / ‘top down’ solution seems to be the most promising.

Top down measures, for instance, might include those that aim at structural or environmental changes, such as might come from funding agencies or by focussing on required training (Korbmacher et al., 2023). Some practical, top down solutions could be the implementation of rules and guidelines by employers or other governing bodies. Meanwhile, bottom up solutions are often much fuzzier. This is in part because they seek to deal with questionable research practices and fraud at the level of researcher behaviour. But to look at behaviour means we also need to consider individual values and motivations, which can be complicated. For instance, such accounts might necessitate implicit or explicit characterisations of what a good, fair, or ethical academic should be or should do, which can result in over-simplified or idealistic, normative accounts of those behaviours.

In the rest of this paper, I explore these issues in more detail and present three key arguments. First, that norms and values promoted as bottom up solutions to the replication crisis rely, to some extent, on sufficient uniformity across humans and their behaviour. Yet we will struggle to find uniformity in either motivations or tendencies. Second, it follows that guiding principles like codes or entreaties to *do better* in science specifically, or academia generally, may motivate those for whom values like integrity are not optional. They can therefore serve to add additional burdensome scrutiny to those who are already committed to these approaches. Meanwhile those with motivations antithetical to integrity are just as likely to find new ways around such guidance. Third, and as a result of these first two premises, bottom up solutions will only work where top down measures are robust and effective. To achieve this last outcome I suggest we need an urgent rethink of some now ubiquitous academic values such as those that prioritise tendencies like ambition, and which use vague measures like ‘excellence’, while promoting hyper-competitive environments that contribute to problematic measures of ‘success’ in academia. Some of this is already happening, including in schemes like the Recognition and Reward approach adopted by some Dutch universities.^1^ But without a more systemic culture shift, including on certain pervasive values, these initiatives may achieve only limited change. I begin to tackle this challenge by analysing the term ‘ambition’.

## Ambitious Academics: Values, Motivations, and Tendencies

Ambition is not a simple nor a neutral value. On the one hand we can understand ambition as encapsulating the strength of someone’s desire or determination to do or to achieve something. In these respects ambition can be considered similar to *aspiration*, or even more banally, as encapsulating someone’s goals, objectives, and plans. Showing oneself as ambitious might therefore be considered akin to showing what is wanted from life and/or a career. For these reasons, ambition is sometimes treated as synonymous with a calling or vocation. But ambition can invite interpretations that go beyond this. The idea of a *desire*, for instance, implies a future projection, such as for future success. This in turn can be tied to a wish or a hope for something, regardless of whether this is something that is in reach. At the extreme it can encompass reward seeking behaviours for that which is not yet achieved, or even for things that will not be achievable. As well as providing motivation and inspiration to do well, ambition can also lead a person to seek that which they have not earned and do not merit. When this happens, ambition may lead someone towards greedy, selfish, or otherwise self-serving behaviours.

Pettigrove (2007) says that philosophers have been split on the question of whether ambition is a virtue or a vice, but suggests that many would consider it a vice with only minor value. Yet this is out of keeping with academic spaces that nurture and encourage ambition, as I describe in this paper. Pettigrove (2007, p. 65) concludes that context is essential for distinguishing between ambition as vice or as virtue:

If ambition is for a good object whose goodness is properly seen, if it is motivated by an appreciation for the object’s value, is pursued through unobjectionable means, generates a desirable outcome and is meaningful, then its virtue will be clear. If it is for a bad object that is poorly understood, is motivated by a lack of self-esteem, anxiety, envy, selfishness, or invidious comparison, brings pain to the agent and those with whom she has to do, is pursued by debased means and fails to be meaningful, then its viciousness will be apparent.

The kinds of distinctions drawn above can also be found in a 2021 study exploring ideas of success and integrity in research, where interviewees considered ‘ambition, passion, and tenacity’ as key elements for success ‘while also arguing that hyper-ambition or excessive desire to be successful could bias conclusions and encourage researchers to loosen their integrity’ (Bonn and Pinxten, 2021).

In this section I argue that in the context of the replication crisis, we should lean towards viewing ambition as a vice more than a virtue. I suggest that the typically individualistic nature of ambition, plus its uncertain qualities, coupled with a tendency to over-value or over-estimate its positivity in academic reward-centred contexts, has led it to be an essential contributing factor to the replication crisis. In other words, a focus on ambition in academia, instead of, say, recognition, has encouraged academics to hyper-fixate on possible, speculative future successes (ends), the achievement of which is then considered (by some, maybe many) to be more important than the path by which someone arrives (means).

For these reasons, I also suggest that terms like *over*-ambition (cf. Annas, 2021) do not go far enough. Instead, by affixing the word ‘hyper’ to ambition, I intend to capture the ways in which ambition may *dominate* someone’s motivations, tendencies, or behaviour such that it becomes primary or all-encompassing. This would at least help to explain how it might overrule other motivations and virtues and lead someone, at the extreme end, to commit fraud and thereby expose themselves to censure. As Wilson (2020, p. 33) notes, the outcomes and punishments if caught undertaking problematic actions can be severe, but for ‘the honourable academic’ the very act of being caught would itself be a shameful punishment.

Thus ‘hyper’ aims to distinguish the description of ‘bad’ or problematic ambition, as outlined in the above account, from what might be called ordinary ‘goal’ or ‘aspiration’ level ambition. This does not imply that the distinction is necessarily easy to draw, nor even that such instances will always be extreme or obvious. Rather it is to acknowledge that ‘ordinary’ ambition may not itself be the cause of bad behaviour, for instance where motives are good, objects worthwhile, or where it occurs in low levels and is compatible with integrity and other virtues. Hyper-ambition should make this distinction clear.

To explain how I arrive at the conclusion above, we can consider another possible antonym to ambition. Annas (2021 p. 25), who acknowledges that ambition does not have ‘a clear opposite’, suggests that the rejection of ambition could be considered ‘unambition’. To assess this suggestion, let’s consider the person who rejects ambition as a primary motivation and see what this can mean for them. It is certainly possible that this person may be seen as virtuous, especially if they do this because they are unimpressed by some negative outcomes of hyper-ambition in academia. Yet this is not the only way to view their behaviour. For some, a rejection of ambition suggests that a person has low standards for their own improvement, which Annas (2021) also describes. While ambition presents go-getting aspirational qualities, a rejection of it seems to suggest stagnation or mediocrity. Meanwhile, *unambition*, as Annas describes it, and *rejection*, as I describe it, are strange antonyms to ambition. The former is not even recognised by my spellcheck, and I concede the latter is rather unusual. It is more common to describe someone who does not demonstrate ambitious behaviours by saying they *lack* ambition – which is generally disparaging. This tells us something important about what we expect. To lack is rarely a good thing (consider the lacking of humility, comfort, or success). The terminology is not neutral, and it directs us in how to position ourselves in relation to the term.

By applying this term, or its opposite (whatever we settle on), to a person, we further imply that they ought to be *somewhere* on the ambition continuum. Yet if a person does not want to identify with the label ‘ambitious’, what will others understand by this action? That they have no goals and aims, or worse, that they are unmotivated and without aspiration? Or can we understand the rejection as a moral one, towards collaborative rather than individual success, for example? I suggest that some current academic evaluative methods tend towards the former interpretation, whereas I suggest it could be either. Ambition might just be a poor catch all term that fails to tell us very much at all. Especially if it leads to conclusions like the one reached by Annas (2021, p.32) that the ‘aspiration to excel’ is ‘something not found “in most people” (at least in most societies and cultures)’. I would suggest instead that *aspirations* to excel may simply be missed when grouped with a highly general and somewhat polarising term like *ambition*.

For instance, a person’s *drive*, which ambition might capture, can also be found in other terms that are not so tied to individually-motivated descriptions. For instance, Annas (2021) suggests that *tenacity*, *determination* and *aspiration* are common elements of ambition. Yet these terms are not dependent on ambition for meaning. Instead, the person who fights for others is tenacious, the person who persists when students consider giving up shows determination, while the person who seeks recognition for their hard work aspires to have their achievement rewarded. These are not mundane actions, and it is not difficult to think of these attributes as coming from *the will to excel*: to excel as defender, teacher, as worthy. Not one of these examples requires reference to ambition in order to be meaningful. Yet such actions can be easily overshadowed by the ostentatious, overstated, or reward-seeking behaviours of those who are hyper-ambitious. In the next section I consider other ways to move beyond thinking about individual ambition, and suggest that the replication crisis makes this task essential.

## The Replication Crisis: Who Cares about Integrity

Some proposed bottom up solutions to the replication crisis include endorsing scientific integrity, ethics, values, and shared norms (Ritchie, 2020),^2^ promoting ‘moral character’ (Andreoletti, 2020), encouraging humility (Jasanoff, 2003, 227; Nerlich, 2013), fostering trustworthiness (Peels and Bouter 2021), and challenging the view of scientists as neutral by acknowledging subjectivity (Kueffer and Larson 2014). In this section I argue that these efforts will only succeed unaided where they are sufficiently meaningful to the person whose motivation we seek to affect. Furthermore, I suggest that hyper-ambitious people are one cause of the replication crisis, perhaps an essential one.

If someone values integrity over success, then we can expect a lower likelihood of transgression, e.g. through questionable research practices like corner-cutting, for instance by not being thorough, or even by being sloppy (Bouter et al., 2016). We can also expect that they are less likely to seek to benefit from what they are not owed. Meanwhile, someone who wants to succeed *no matter what*, or above all else, is more likely to do these kinds of questionable things. I qualify this last sentence with a ‘more likely’ because it is certainly not a given that someone who is hyper-ambitious will do shoddy work or commit fraud, say. However, where someone acts of their own free will (is not coerced or bullied), has the power to choose and to decide (they are not otherwise being led), and intentionally cuts corners or commits fraud, then it is not unreasonable to expect this to correlate to some extent with a deficit in their integrity, ethics, or whichever values or terms we choose from the above list. But regardless of whether we consider this deficit a moral failing or something else, it seems to me that a person who values integrity would seek to avoid such behaviours (see also Wilson, 2020).

There will be plenty of exceptions to the above account that we ought to briefly consider at this juncture. It is possible, for instance, that hyper-ambition is fuelled by a fear of failure, rather than a desire for particular success, and in these respects it may encompass a wish to be considered with sufficient or equal regard to others, or not to be left behind (Pettigrove, 2007). We must also take into account other kinds of pressures and influences that a person faces in their working life. As well as the broader pressures that come from being in a competitive environment like academia, there are financial contexts to consider: there are few jobs, much precarity, and a growing number of newly qualified academics keen to join the academy. Hyper-ambition thrives in this environment, since it tends to encompass competition (Pettigrove, 2007). I suspect we might define these responses in ways that do not require reference to ambition, but for the purposes of this paper I am willing to concede that ambition can play a role.

Someone can also be ambitious primarily in relation to their own current state, but this does not preclude comparison to others. Especially in academia, where competition is actively encouraged, and where some kinds of success necessitate comparison: it is only meaningful to be top of your field if your field encompasses more than you. Where someone plays with this kind of ambiguity to their advantage, for instance by claiming to be at the top of a field where they are in fact alone, we can more easily dismiss this as misleading. In addition, since ambition (hyper or otherwise) may not be easily satiated – there is always more to achieve – we can expect this competition to persist beyond fulfilling fundamental needs like job security. Hyper-ambitious people with tenure, seniority, and success are just as likely to seek further reward – we may never see a dip in someone’s ambition (see also Ritchie, 2020).

There is more that could be said on all of this, but instead let us return to the case of Toni and Sophie that I outlined above. My description of these academics sought to avoid some of the more problematic, autonomy-limiting contextual and environmental issues that we just considered. In Toni and Sophie we have motivations that are personal and professional: their jobs are not at risk (no more than anyone else’s) and there is nothing in the account to indicate that they *need* the promotion they each seek beyond their own aspirations. That other motives and tendencies exist is not in question, but in the case of Toni and Sophie, we can at least have some confidence in their respective autonomy, decision making, and judgments. This is important because my intention in this paper is to explore what can arise because of different values, motivations, and tendencies as displayed in actions, not to interrogate the deeper reasons for those values, motivations or actions, including by way of a deep psychological assessment of these hypothetical academics. It could be reasonable to assert that such tendencies can also be dispositional, by which I mean somehow part of someone’s character or being, but to explore this in more depth would go beyond the scope of this paper. In the case covered here, it is sufficient if we can expect their choices and motives to be *sufficiently* their own.

Toni’s lack of scruples, integrity, or however we describe her actions and related, is, I suggest, tied to her hyper-ambition, and it has proven to be a distinct advantage in her path to academic success (see also Ritchie, 2020). We could, if we wanted, add to this many other tricks that she could fruitfully employ. For instance, it is easier to get work to publication quickly and efficiently if you cut corners (Bouter et al., 2016). The same applies where researchers exploit grey areas for strategic uncertainty and ambiguity (Heesen, 2018). We might also expect someone like Toni to co-opt legitimate strategies for humility, such as hedging (avoiding undue certainty) to avoid criticism, or to *imply* what will not hold if stated explicitly (Kolodziejski, 2014). Academia too often rewards the scientific outcomes of these behaviours, without due consideration to the path someone follows to achieve them. With the success that results, the hyper-ambitious academic provides a model that others may be encouraged to follow.

For instance, personal success can result in at least some of these hyper-ambitious people gaining powerful academic positions, including as editors and other gatekeepers (Barzilai-Nahon, 2009; Primack et al., 2019). They may also be invited to review and evaluate other colleagues and their work, including those who are junior. As senior colleagues they may be taken as role models, and whether they promote and reward hyper-ambitious tendencies, or whether others are simply inspired by them, we can expect that at least some people may be influenced and seek to emulate their successes as well as their methods. For instance, if someone cuts corners to achieve success, it is reasonable to expect that, under their leadership, the cutting of corners may be tolerated, expected, or otherwise exploited. And since these spaces are largely self-governing, this behaviour may not be known unless someone whistleblows or something forces exposure.

Toni in our thought experiment operated at a relatively low level of questionable practice, such that some may not see her behaviour as problematic. If she continued in that vein it could be that nothing she does will draw negative attention. Her career may never be impacted by any censure, and integrity promoting measures are unlikely to affect her (directly or morally) so long as she continues to receive rewards in real terms. Meanwhile, integrity measures are likely to speak to Sophie, given her tendencies and actions. She may take time to ensure her students understand important integrity initiatives, maybe she joins a committee to develop policies to address suchproblems. The drive to do good easily leads in such directions, even when there is low reward for the time invested.

The final point to consider here is that any measure we offer to improve the situation I have described will come with its own cost. It may, for instance, create new problems at the same time as solving others, or it may be co-opted by those who see no reason not to. For the first issue, we can consider the case to make science accessible, and for the second, the promotion of adversarial engagement. The push for accessible, transparent, or open access science provides a valuable tool to ensure that scientific research remains open to scrutiny. Scientific studies on which political decisions, medical practice, and public policies rely ought to be open to view. Yet the principles of accessibility can cause problems if they are too broadly applied. Work, ideas, or people who are complicated, complex, and nuanced, may not be easy to understand. It does not follow that they should not be valued, but this is too often the result (Erden & Altorf, 2020). This is similar to the question of ‘impact’: we can value impactful research, but it would be problematic to say that *all* research should have an obvious or clear impact. Knowledge for the sake of knowledge can bring value of its own, even if this may not be immediately apparent to contemporary thinkers.

Similarly, ‘adversarial collaboration’ is useful as a tool to ensure academic integrity (Ritchie, 2020). In this way, someone’s work is open to challenge and therefore scrutiny. Yet this too can be a double edged sword. While scepticism may encourage scrutiny, and this is welcome, especially where academics have utilised questionable research practices or been fraudulent (Heesen, 2018), these tools can be appropriated by researchers who will use adversarial tactics in order to be seen as assertive or confident, to take down ‘rivals’, or to silence critics. It seems clear therefore that worthy tools can exacerbate inequalities when reward systems do not deter bad behaviour.

## What can we Change?

In this section I present the case for robust top down measures that would support bottom up solutions. We can begin by reassessing what we value in academics and their research, and how we measure and promote these values. For instance, novelty in some areas of research, and in some fields, may be difficult to achieve: much has already been said or discovered, and it may be rare to have new things to say on a regular basis. Research often builds slowly and tentatively, in order to ensure that each brick in the structure is well formed and reliable. This is not easy to reward. Meanwhile, if researchers need to demonstrate novelty or excellence, this can encourage overstatement, hype, and exaggeration (Moore et al., 2017). There are other good reasons to resist these metrics, of course, including that excellence cannot be measured quantitatively without proxies that must themselves be evaluated qualitatively. For instance, if you measure excellence by reference to a citation index you still need to make a qualitative assessment and thereby judgement about the reliability and value of a selected index. Yet these judgements are too often obscured by statistics and other data that pose as purely objective measures.

We need to consider how we avoid perpetuating the contexts in which researchers may be encouraged to engage in questionable research practices, whether to attain certain goals, or to satisfy (even prioritise) their hyper-ambitions. To achieve this, we would do well to value a plurality of ends and outcomes in research, as well as tendencies in academia, and to think beyond the rhetoric of ambition as purely positive. This means going far beyond paying lip service to diversity. For instance, we need to consider those who do valuable replication work, even where this does not produce metric-attracting novelty^3^ (Andreoletti, 2020).

Similarly, we need to consider the colleague who does not prioritise research and not assume this is because they do not care about it. Perhaps they love teaching and care about students, and this should also be valued. In so doing we should not expect that this person can simply switch to promoting themselves as a superstar teacher. Apart from recognising that some academics will simply not promote themselves very much at all, we also need to consider that teaching metrics will face the same problems as those we see in research: what do they measure, and how do you distinguish between popularity, status, and good teaching? There is also a great risk of bias in such measures (cf. Mitchell and Martin 2018). Similarly, collegiality, being student-centred, or more generally demonstrating *good scientific citizenship* (Ritchie, 2020), is as valuable as, and sometimes more valuable than, individual hyper-ambition. In recognising this, we also need to avoid a situation where these terms become new metrics that simply shift the focus of hyper-competition from one area to another.

To broaden our methods of evaluation means taking into account the various ways that an academic can be considered valuable, including to their institution, their colleagues, and their students. We can achieve this in various ways. For instance, we can encourage people to reach their individual potential rather than to live up to standards that prioritise a narrow strand of motivations and tendencies. In short, the university and its employees will achieve more if we seek to ensure appropriate recognition for a broader variety of academic work than we currently do, whether academics seek this or not. Thinking back to our thought experiment we could consider the promotion prospects of each academic by offering a variety of routes, by taking into account a wider variety of achievements and experiences, and by avoiding measures and metrics, like citation scores, that we already know are problematic, and which suit some academics (Ritchie, 2020) and indeed disciplines (Erden & Altorf, 2020) better than others.

We can take this further in the following three ways. First, by encouraging greater scope for the assessment of the means, as well as the ends, of activities like research, and by including space to consider reasons and actions. Did someone get a large grant because they neglected their other academic duties, leaving junior colleagues to pick up the slack and to face being over-worked in the process? If so, then this behaviour ought to be censured and the value of the reward tempered. Second, we can give greater priority to behaviours and actions that demonstrate consideration, dedication, or commitment to a broader range of academic values than those that are hyper-individualised, or in the service of hyper-ambitious ends. Related to this, there is little that is captured by a polarising term like ‘ambition’ that cannot be covered in other ways, especially where alternatives would not as easily hide greedy, selfish, or other problematic and harmful behaviours. In short, we ought to make these behaviours harder to disguise by avoiding terms that can be easily co-opted. Third, we can promote a broader approach to academic reward that does not simply pay lip service to diversity. Instead it should recognise the value of academics who do not meet current standards of ‘success’ as measured by a term like ambition, or metrics that are quantitative. Without this, integrity initiatives are likely to speak primarily to researchers who are themselves already concerned about such issues, and risk burdening these same people who are already overworked in filling the integrity void created by those who seek to do well for themselves alone. We cannot expect this to change when the systems and structures reward and thereby perpetuate self-serving behaviours.

Some of this may be difficult to achieve, but is not impossible to already find better ways to recognise and value the labour of academics who enjoy and are good at, say, teaching, administration, or other non-metric-attractive, underappreciated, yet valuable aspects of academia. It is easy to see how these kinds of labour may be promoted as especially valuable, perhaps more than those efforts that satisfy only individual hyper-ambitions. From a dedicated teacher who cherishes integrity we are likely to have generations of new researchers encouraged to value integrity. From a dedicated self-server, we may have academics trained to be hyper-ambitious and willing to do whatever it takes to succeed. The replication crisis ought to show us why this is an outcome we should seek to avoid.

## Conclusion

In this paper I have argued that some central features of the replication crisis can be explained by academic measures of success that promote, encourage, and reward hyper-ambition. I suggest that addressing researchers’ values, norms, or motivations as bottom up solutions should not rely on there being sufficient uniformity between people such that we expect ‘catch all’ integrity measures to be effective. Instead I suggest that we pay more attention to hyper-ambition, and the rewarding of this tendency or its outcomes. Otherwise, when we encourage academics *to be better*, we will speak to those for whom integrity is not optional, but largely be ignored by others. For these reasons, bottom up solutions that focus on integrity and similar will only work where top down measures include methods to censure bad behaviour. I include some examples to show what these could be, but there are many more to consider. Context matters if we are to get to the heart of what we want the academy to be, to do, and to change in the light of the replication crisis.

This paper has argued that if we truly want academics *to do better*, then we need to provide good models that demonstrate the recognition and reward of good or positive *behaviour* in the academy as well as in research. This recognition should count for at least as much as, sometimes more than, existing methods for success measurement (publications, grants, accolades etc.). If we do not challenge the status quo that continually rewards outcomes from a range of questionable research practices, we should not expect the new generations of researchers to easily avoid these temptations. Why should they, when they see where others do not, and where those others are rewarded? This is, I argue, especially the case for those who already tend towards hyper-ambition when they enter the academy.

To illustrate this, I presented a thought experiment of two academics, Toni and Sophie. In a system with a wider approach to what is valuable, we create scope for Sophie to receive recognition and reward. Meanwhile, at the early stages of Toni’s career, her ambition can be fostered in various ways, including to positive ends. But where the categories to measure success are not amended, we can simply expect more of the same: to achieve further promotions, Toni may overstate the excellence of her work, and to achieve a big grant she may make false claims about novelty. As she becomes more senior, she may make liberal use of junior colleagues’ time without much care for their needs or prospects beyond her direct interests. None of this behaviour is especially unusual, and little would be sufficiently or obviously problematic such that she would necessarily face substantial challenges to her behaviour. Toni is too common and too ordinary to stand out as particularly bad, while Sophie may not achieve the success necessary to develop a platform from which to challenge the status quo. Yet without such challenge, we cannot hope to counter the hyper-competitive environments that foster hyper-ambitious academics who in turn liberally contribute to the replication crisis.

## Data Availability

Not applicable.

## Footnotes

1 More information here: https://recognitionrewards.nl/about/about-the-programme/.

2 Such as ‘Mertonian Norms’. Outlined in Ritchie (2020, 21), these broadly encompass (1) universalism, where methods have priority, (2) disinterestedness, over ideology or reward, (3) communality, whereby knowledge is shared, and (4) organised scepticism, such that all should remain open to scrutiny.

3 One helpful reviewer has pointed out that replications and replication targets can themselves be, or at least be made to be, ‘metrics attractive’. For instance with increased funding for, say, large-scale collaborations that in turn produce landmark replications of the kind generated by the Open Science Collaboration (2015). They suggest that this kind of grant, with associated benefits (large teams, high impact articles), could provide ethical pathways with similar incentives, including for hyper-ambitious researchers. Since, as I note previously, the negative outcomes I identify with hyper-ambition are not a given, this approach could be effective indeed. That said, it is not a given that additional (ethical) incentives will address the full range of harms that I suggest can come from hyper-ambitious tendencies and behaviours. The problem is not simply structural; it is also about the tendencies of those people who have flourished within such structures and why, and thereby requires a broader range of considerations when trying to achieve different ends.
